# Homotopic non-local regularized reconstruction from sparse positron emission tomography measurements

**DOI:** 10.1186/s12880-015-0052-5

**Published:** 2015-03-18

**Authors:** Alexander Wong, Chenyi Liu, Xiao Yu Wang, Paul Fieguth, Hongxia Bie

**Affiliations:** Department of Systems Design Engineering, University of Waterloo, Waterloo, Ontario Canada; Department of Information and Communication Engineering, Beijing University of Posts and Telecommunications, Beijing, China

## Abstract

**Background:**

Positron emission tomography scanners collect measurements of a patient’s *in vivo* radiotracer distribution. The system detects pairs of gamma rays emitted indirectly by a positron-emitting radionuclide (tracer), which is introduced into the body on a biologically active molecule, and the tomograms must be reconstructed from projections. The reconstruction of tomograms from the acquired PET data is an inverse problem that requires regularization. The use of tightly packed discrete detector rings, although improves signal-to-noise ratio, are often associated with high costs of positron emission tomography systems. Thus a sparse reconstruction, which would be capable of overcoming the noise effect while allowing for a reduced number of detectors, would have a great deal to offer.

**Methods:**

In this study, we introduce and investigate the potential of a homotopic non-local regularization reconstruction framework for effectively reconstructing positron emission tomograms from such sparse measurements.

**Results:**

Results obtained using the proposed approach are compared with traditional filtered back-projection as well as expectation maximization reconstruction with total variation regularization.

**Conclusions:**

A new reconstruction method was developed for the purpose of improving the quality of positron emission tomography reconstruction from sparse measurements. We illustrate that promising reconstruction performance can be achieved for the proposed approach even at low sampling fractions, which allows for the use of significantly fewer detectors and have the potential to reduce scanner costs.

## Background

The concept of positron emission tomography (PET) imaging revolves around the measurement of a patient’s *in vivo* radiotracer distribution. The system detects pairs of gamma rays emitted indirectly by a positron-emitting radionuclide (tracer), which is introduced into the body via biologically active molecules. As such, PET imaging can be described via a line-integral model of acquisition [1]. PET data are collected as projections in sinograms or listmode format [2]. The raw data collected by a PET scanner are a list of ‘coincidence events’ representing near-simultaneous detection, each coincidence event represents a line in space connecting the two detectors along which the positron emission occurred. While one can employ the inverse Radon transform to recover tomograms from acquired PET data, such an approach can be unstable, particularly when dealing with noise-contaminated data [3]. Therefore, the filtered back projection algorithm, which is a stabilized and discretized version of the inverse Radon transform, is commonly used in practice [3].

There are two common approaches for tomographic reconstruction: i) filtered back projection (FBP) [4], and ii) iterative expectation-maximization (EM) [5-7]. FBP has been widely used to reconstruct tomograms from the projections in clinical settings due to its overall simplicity and low computational complexity. However, shot noise in the raw data is prominent in the reconstructed tomograms and areas of high tracer uptake tend to form streaks across the tomogram.

Iterative algorithms for reconstructing tomograms from acquired PET data take into account the statistical nature of the acquired projection data and incorporate the physical model into reconstruction. These algorithms compute an estimate of the likely distribution of annihilation events that led to the measured data, based on statistical principles [8]. The advantage is a better noise profile and resistance to the artifacts common with FBP, but are typically more computationally intensive.

Recently, wavelet-based methods [3,8-10] have also been proposed for solving this inverse problem in PET reconstruction. Wavelet-based methods have been applied in research literature in the post-processing stage to suppress artifacts in the reconstructed tomograms [3]. Another attractive approach to reconstructing tomograms from PET data using wavelets is to incorporate wavelets into the reconstruction process itself [9], where an *L*_1_-regularization constraint is used to enforce sparsity in the wavelet domain. For example, wavelet regularization using exponential-spline wavelets has been shown to produce good reconstruction results [10].

More recently, the concept of compressive sensing [11] has offered great potential for signal recovery with very high accuracy through sparse measurements acquired at very low sampling fractions. Researchers have also begun to explore the use of compressive sensing for image reconstruction in various medical modalities [12]. To date, most sparse medical image reconstruction algorithms have focused on solving the sparse reconstruction problem via *L*_1_ minimization.

The main contribution of this paper is to introduce and investigate the potential of a homotopic non-local regularization (HNLR) reconstruction framework for the reconstruction of PET tomograms from sparse measurements, with the aim to achieve high reconstruction quality. The ability to achieve strong reconstruction results at low sampling fractions can be very helpful for reducing the number of detectors needed in a PET system [7], which in effect helps can lead to reduced scanner costs and potentially improve adoption of PET in developing countries.

The rest of the paper is organized as follows. First, the underlying methodology behind the proposed use of a homotopic non-local regularization (HNLR) reconstruction framework for the reconstruction of PET tomograms from sparse measurements is described in Section “Methods”. The experimental results are presented in Section “Results and discussion”. Finally, conclusions are drawn and future work is discussed in Section “Conclusion”.

## Methods

The underlying methodology behind the proposed approach to reconstructing PET tomograms from sparse measurements can be explained as follows. The underlying imaging system can be represented with the following linear relationship

(1) $$ P(\alpha,s)=\mathbb{R}{f(x)}   $$

where *P* is the set of sampled measurements, *s* is the distance of straight line *L* from the origin, *α* is the angle that the normal vector to *L* makes with the *x* axis,  is the Radon transform, and *f* is the unknown tomogram. The goal of reconstruction is to use the sampled measurements *P* (projections in the Radon transform domain through the object) to find the tomogram *f* in image space.

### *L*_*p*_ minimization for sparse reconstruction

In PET, the number of projections is related to the number of detectors. Therefore, taking a high number of projections requires tightly packed discrete detector rings [7], which could lead to high scanner costs. Instead, our goal is to measure only a fraction of the projections, hence reducing the number of detectors needed, and still be able to get high quality reconstructions. Consequently, Eq. 1 should be rewritten as:

(2) $$ f(x)=\mathbb{R}^{-1} \left \{ \Phi P(\alpha,s) \right \}   $$

where $\mathbb {R}^{-1}$ is the inverse Radon transform operator and *Φ* is a measurement operator defining which of the sites are measured (non-measured projection space indices will be assigned 0 by *Φ*). Our goal is to reconstruct *f*(*x*) from a sparse sampling of *P*(*α*,*s*). With only few measurements made, not all sites are measured, making this problem an ill-posed inverse problem [13], and as such there may exist multiple solutions. A common method to solving this inverse problem is through the use of *L*_2_ minimization:

(3) $$ \hat{f}(x) \in \underset{f(x)}{arg\, min} \left \|\, f(x) \right \|_{2}\; \; s.t. \; \mathbb{R}{f(x)}=\Phi P(\alpha,s)   $$

where $\hat {f}(x)$ is the estimated signal in image space, and $\hat {P}(\alpha,s)$ is the estimated signal in projection space. However, solving the problem in this manner results in significant artifacts such as aliasing and blurring in practical situations [12].

Recently, scientists realized that many signals, such as PET imagery, possess an inherent sparsity in some sparsifying transform domain [14]. According to the emerging theory of compressive sensing [11,15], a better estimate of *f*(*x*) can be obtained by maximizing the sparsity of the signal in the transform domain and enforcing data fidelity in projection space domain. One can formulate the reconstruction problem as a constrained *L*_0_ minimization problem,

(4) $$ \hat{f}(x) \in \underset{f(x)}{arg\, min} \left \| \Psi f(x) \right \|_{0}\; \; s.t. \; \mathbb{R}{f(x)}=\Phi P(\alpha,s)   $$

where *Ψ* is the sparse transform operator. Two commonly used sparse transform operators are the finite differential transformation and the wavelet transformation [16].

Unfortunately, solving the *L*_0_ problem is essentially NP hard, and as such is intractable in practice [17]. To address this problem, pioneering work done by Candes [11] and Donoho [15] showed that under certain conditions, *L*_0_ and *L*_1_ minimization can lead to the same solutions. As such, one can instead solve the *L*_1_ minimization as:

(5) $$ \hat{f}(x) \in \underset{f(x)}{arg\, min} \left \| \Psi f(x) \right \|_{1}\; \; s.t. \; \mathbb{R}{f(x)}=\Phi P(\alpha,s)   $$

Theoretically, under certain conditions, solving the *L*_1_ problem can get exactly the same solution as solving the *L*_0_ problem, although a substantial increase in the number of measurements is required [12,18].

Typically, a total variation (TV) penalty [19] is employed in the *L*_1_ minimization framework, which is known to have an edge-preservation effect, and to account for the unavoidable noise in the measurements, the data fidelity constraint is typically replaced by a *L*_2_ norm constraint:

(6) $$ \begin{aligned} \hat{f}(x) \in \underset{f(x)}{arg\, min} \left \| \Psi f(x) \right \|_{1}+TV\left(f(x)\right)~~~~ \\ s.t. \; \left \| \mathbb{R}{f(x)}-\Phi P(\alpha,s) \right \|_{2}<\epsilon  \end{aligned}\vspace*{10pt}  $$

### Homotopic, non-local regularization based reconstruction

There are two fundamental limitations associated with the reconstruction framework described in Eq. 6 for the purpose of reconstructing tomograms [20]. First, the use of a TV penalty can lead to the loss of fine structure at low sampling fractions [12]. Since PET images are characterized by complex functional process variations, the TV approach may not be well-suited for reconstructing these data. Second, as already mentioned, the number of measurements required for solving the *L*_1_ problem can be noticeably higher than that for the *L*_0_ problem, which is undesirable as it leads to an increased number of detectors needed. Hence, an alternative reconstruction strategy that addresses these two problems is desired.

To achieve the theoretical capability of the constrained *L*_0_ minimization approach without a drastic increase in the number of measurements, Trzasko et al. [12] introduced a homotopic *L*_0_ minimization framework, which uses an increasing approximation framework to get close to the *L*_0_ minimization problem:

(7) $$ \begin{aligned} \hat{f}(x) \in {\lim}_{\sigma \to 0} \underset{f(x)}{arg\, min} \; \rho (|\Psi f(x) |,\sigma) \\ s.t. \; \left \| \mathbb{R}{f(x)}-\Phi P(\alpha,s) \right \|_{2}<\epsilon \end{aligned}   $$

where *ρ* is the homotopic approximation of the *L*_0_ norm, which approaches the *L*_0_ norm as *σ* approaches 0. Experimental results showed that this strategy is able to approach the capabilities of the *L*_0_ minimization approach [21], thus addressing the problem pertaining to the number of measurements required.

To address the problem of detail loss from using total variation, we instead integrate the concept of non-local regularization, often used in image processing for improved detail preservation [19,20,22-24], into the homotopic *L*_0_ minimization framework [25] for the purpose of tomogram reconstruction from sparse PET measurements, as it is well-suited for handling fine structural details. The proposed homotopic, non-local regularization framework for sparse PET reconstruction is formulated as follows:

(8) $$ \begin{aligned} \hat{f}(x) \in {\lim}_{\sigma \to 0} \underset{f(x)}{arg\, min} \; \eta (|\Phi f(x) |,\sigma) \\ s.t. \; \left \| \mathbb{R}{f(x)}-\Phi P(\alpha,s) \right \|_{2}<\epsilon \end{aligned}   $$

where *η* denotes the homotopic non-local regularization functional:

(9) $$ \eta(|\Phi f(x)|,\sigma)=\left [\sum\limits_{\textit{x}\in\Omega} \sum\limits_{y\in N(x)}w(x,y,\sigma) \cdot (N(x)-N(y))^{2} \right]   $$

*σ* controls the approximation degree, and *w*(*x*,*y*,*σ*) is defined as

(10) $$ w\left [ x,y,\sigma \right ]=exp\left \{ -\frac{(N(x)-N(y)}{2\sigma^{2}} \right \}   $$

where *N*(*x*) is a neighborhood around *x*. Essentially, the idea is to minimize the homotopic non-local regularization function, denoted by *η*(|*Φf*(*x*)|,*σ*), at decreasing values of approximation degree, denoted by *σ*, such that *η* approximates the *L*_0_ norm closer and closer. An approximate solution of Eq. 8 can be obtained using an iterative optimization approach, with decreasing values of *σ* as the number of iterations increases. To reduce the computation complexity, the neighborhood search space is limited to a window search around the pixel to be estimated. The regularization term *η*(|*Ψf*(*x*)|,*σ*) is designed to efficiently suppress artifacts associated with incomplete measurements while preserving detail and structure, and is enforced via steepest descent [26,27], while the data fidelity term is designed to ensure that the reconstructed tomogram complies with sampled measurements while accounting for some level of noise artifacts. The data fidelity term is enforced at measured projection space indices at each iteration *i* as follows:

(11) $$ {\fontsize{7.8pt}{9.6pt}\selectfont{\begin{aligned} &P_{i}(\alpha,s)\\ &\,=\,\left\{\!\!\begin{array}{ccccc} \mathbb{R}\,{\hat{f_{i}}(x)} &\! \text{if} &\! \left \| \mathbb{R}{\hat{f_{i}}(x)}-\Phi P(\alpha,s) \right \|_{2}\!\!<\epsilon\\ P(\alpha,s)+\left(\!2H\left(\!\mathbb{R}\,{\hat{f_{i}}(x)}-\Phi P(\alpha,s)\!\right)-1\!\right)\frac{\epsilon}{b} & \!\text{if} & otherwise\\ \end{array} \right.  \end{aligned}}}  $$

where *H*(.) is the Heaviside step function and *b* is the number of measured projection space indices. The pseudocode for the proposed homotopic non-local regularized reconstruction method is shown in Algorithm 1. Note that a *L*_2_ data term is used here for simplicity in the iterative optimization realization shown in Algorithm 1, and more advanced data terms such as the Kullback-Leibler divergence [28,29], which takes better advantage of the Poisson noise statistics of PET, may be used.



### Experiments

#### Data description

To evaluate the effectiveness of the proposed method, sparse reconstructions were performed using a simulated phantom generated using ASIM [30] at 30% sampling fraction (defined here as the percentage of projection angles from which measurements are obtained). The simulated phantom consists of 4 spots of different sizes and is useful for observing the effects of reduced sampling on spatial resolution. Also, since the simulated phantom generated using ASIM is contaminated by simulated Poisson-distributed noise, it is also useful for observing the performance of the proposed method when faced with noisy data. Since the total number of projection angles used in this study is 180 projection angles, that means at a 30% sampling fraction the number of projection angles used for sparse reconstruction is 54 projection angles. Also, the object covers approximately 70% of the field of view. Furthermore, sparse reconstructions at different sampling fractions were performed using three real PET data sets hosted by Harvard Medical School [31], in accordance with Harvard Medical School ethics procedures. The PET data sets are acquired with Fluorine-18 Deoxyglucose (FDG) as the biologically active molecule. The first PET data set (PET1) is acquired from a 73 year-old man who sought medical attention due to a grand mal seizure and progressive speech difficulties, with a brain biopsy revealing grade II astrocytoma. The second PET data set (PET2) was taken from a 53 year-old man who sought medical attention due to a grand mal seizure, with a brain biopsy revealing grade IV astrocytoma. The third PET data set (PET3) is acquired from a 70 year-old man with mild Alzheimer’s disease about 9 months prior to imaging. The PET data sets are 256×256×23 voxels. The data sets were forward-projected from a previous reconstruction obtained via filtered back-projection (FBP) [2] based on measurements from 100% of the projection angles in order to obtain projection data for evaluation.

#### Implementation

For comparison purposes, the proposed homotopic non-local regularization (HNLR) algorithm was compared with the standard filtered back-projection (FBP) algorithm [2], as well as the more advanced iterative expectation maximization algorithm with total variation regularization (TVR) algorithm [6]. The standard FBP algorithm was chosen for baseline comparisons as it is widely used in clinical settings for its speed and ease of implementation. The TVR algorithm was chosen as it is one of the more advanced reconstruction approaches available for PET reconstruction [6,7]. To allow for quantitative assessment, the signal-to-noise ratio (SNR) was computed for reconstructed data at different sampling fractions. Furthermore, a qualitative visual assessment is performed on the reconstructed data to investigate the reconstruction performance and the preservation of details achieved using the tested methods. Different sampling fractions were achieved by sampling the projection angles at evenly spaced intervals. For example, to achieve a 50*%* sampling fraction, every other projection angle from the set of total projection angles was sampled. This methodology was used for all the data sets and the comparison of all three methods. For this study, the parameters of the HNLR algorithm were set as follows as it yielded strong reconstruction performance. The neighborhood *N*(*x*) was chosen as a 9×9 neighborhood around *x*, *σ*_1_ and *σ*_*t*_ are set as 20% and 1% of the dynamic range in image space, respectively, *λ* is set as 0.8, *ε* is set as 2% of the dynamic range in projection space multiplied by the number of measured projection space indices (*b*), and *n* is set as 100 as these parameters were found empirically to provide strong reconstruction performance. Note that the parameters of the HNLR algorithm can be tuned based on the PET imaging system the algorithm is incorporated into to obtain optimal performance for clinical data obtained from the PET system.

### Results and discussion

In order to perform a comprehensive and systematic assessment of the reconstruction performance of the different methods, the peak signal-to-noise ratio (PSNR) was computed for a wide range of sampling fractions. The PSNR metric was computed as follows:

(12) $$ PSNR=10 \cdot {log}_{10}\left (\frac{max(x)^{2}}{MSE} \right)   $$

and (MSE) was defined as mean squared error between original image and reconstructed image:

(13) $$ MSE=\frac{1}{N}\sum\limits_{\textit{x}\in\Omega}\left(f(x)-\hat{f}(x)\right)^{2}   $$

where *f*(*x*) is original image, $\hat {f}(x)$ is reconstructed image, and *N* is the number of pixels in each image.

**Figure 1:**
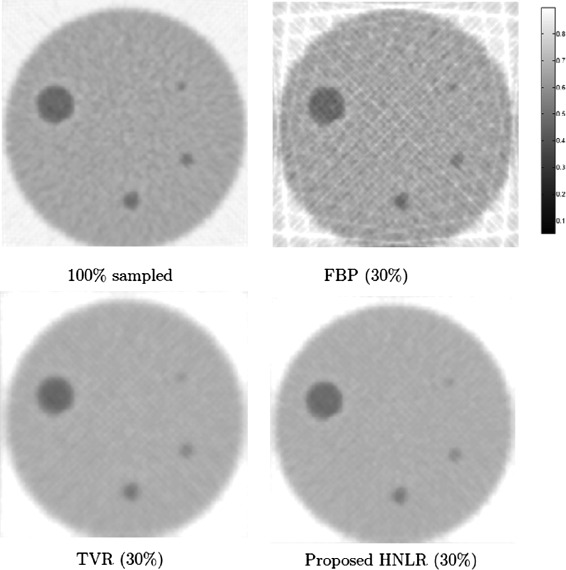
**Reconstructed tomograms from simulated phantom using three different reconstruction methods based on 30% sampling.** We can see very clearly that the FBP method leads to considerable artifacts in the reconstructed tomogram, with significant streaking artifacts over both the background and the phantom details. Both TVR and HNLR methods were able to significantly suppress these artifacts caused due to sampling at sub-Nyquist sampling fractions. It can also be observed that the details (e.g., dark circles) in the tomogram reconstructed using HNLR appear sharper than that reconstructed using TVR.

Figure 1 shows PET tomograms for the simulated phantom reconstructed at 30% sampling using the three reconstruction methods. We can see very clearly that the FBP method leads to considerable artifacts in the reconstructed tomogram, with significant streaking artifacts over both the background and the phantom details. Both TVR and HNLR methods were able to significantly suppress these artifacts caused due to sampling at sub-Nyquist sampling fraction. It can also be observed that the details (e.g., dark circles) in the tomogram reconstructed using HNLR appear sharper than that reconstructed using TVR. Furthermore, it can be observed that while the reconstructed PET tomograms produced by the reconstruction methods are able to resolve all 4 spots in the phantom, the resolution of the smallest spot noticeably suffers as a result of the reduction in sampling and illustrates the fundamental tradeoff between spatial resolution and sampling fraction. For quantitative evaluation, the PSNR for the PET tomograms reconstructed using FBP, TVR, and HNLR are calculated as 8.03 db, 38.48 dB, and 38.84 dB, respectively.

**Figure 2:**
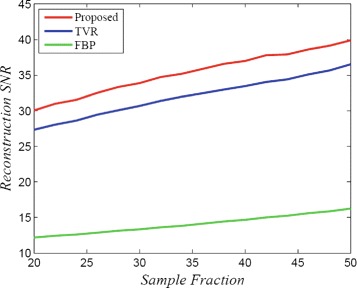
**Average SNR of three different reconstruction methods across PET sets, as a function of sampling fraction.** The proposed HNLR method produces reconstructed PET data with higher SNR values for all levels of sampling fraction when compared to the FBP method and the TVR method.

The average PSNR of the reconstructed PET data sets is shown in Figure 2, as a function of the sampling fraction (i.e., the percentage of projections sampled used to perform sparse reconstruction). It can be observed that the proposed HNLR method produces reconstructed PET data with higher PSNR values for all levels of sampling fraction when compared to the FBP method and the TVR method. It is important to note that there is no fundamental tradeoff in terms of spatial resolution as the data fidelity in projection space is preserved in the proposed HNLR method.

**Figure 3:**
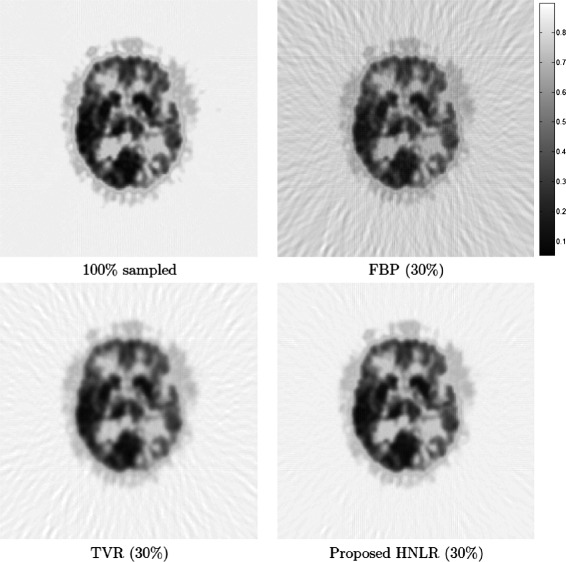
**Reconstructed tomograms from the PET1 data set using three different reconstruction methods based on 30% sampling.** The PET1 data set was taken from a 73 year-old man who sought medical attention due to a grand mal seizure and progressive speech difficulties, with a brain biopsy revealing grade II astrocytoma. It can be observed that the reconstructed tomogram using the FBP method exhibits the most significant artifacts (both in the background area and the imaged brain area), when compared to TVR and the proposed HNLR methods. The reconstructed tomogram using the TVR method exhibits significantly reduced but still noticeable artifacts in the background area, and no artifacts in the imaged brain area. However, there is noticeable loss in fine detail in the imaged brain area. Finally, the reconstructed tomogram using the proposed HNLR method exhibits the least amount of artifacts in the background area when compared to FBP and TVR, no artifacts in the imaged brain area, and noticeably better preservation of fine detail in the imaged brain area.

**Figure 4:**
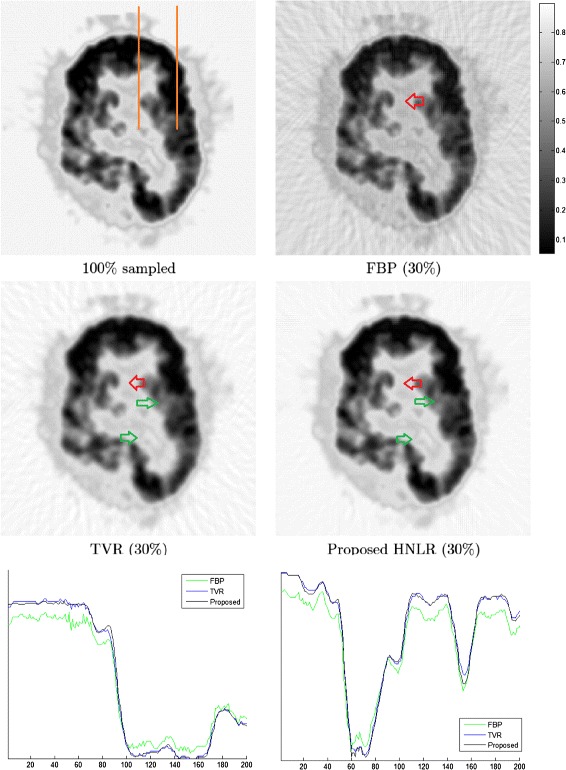
**Reconstructed tomograms from the PET2 data set using three different reconstruction methods based on 30% sampling.** The PET2 data set was taken from a 53 year-old man who sought medical attention due to a grand mal seizure, with a brain biopsy revealing grade IV astrocytoma. As with the reconstructed tomograms from the PET1 data set, it can be observed that the FBP method results in the most significant artifacts in both the background and imaged brain areas when compared to TVR and HNLR, the TVR method results in reduced artifacts but noticeable loss in fine detail in the imaged brain area, and the HNLR method results in the least amount of artifacts and better preservation of fine detail in the imaged brain area. It is very clear that streaking artifacts (caused by sampling at a sub-Nyquist sampling fraction) is prominently overlaid on the entire brain region in the reconstruction produced using the FBP method (see red arrows), while they are significantly reduced in the reconstructions produced by TVR and HNLR. It is also important to highlight that, within the same brain region in the HNLR reconstruction, there is an overall improvement in sharpness when compared to the TVR reconstruction (see green arrows). Two line profiles (indicated by orange lines) from the PET data sets reconstructed using the methods are shown at the bottom, and further illustrates the reduction in artifacts and improved contrast achieved by TVR and HNLR when compared to FBP.

**Figure 5:**
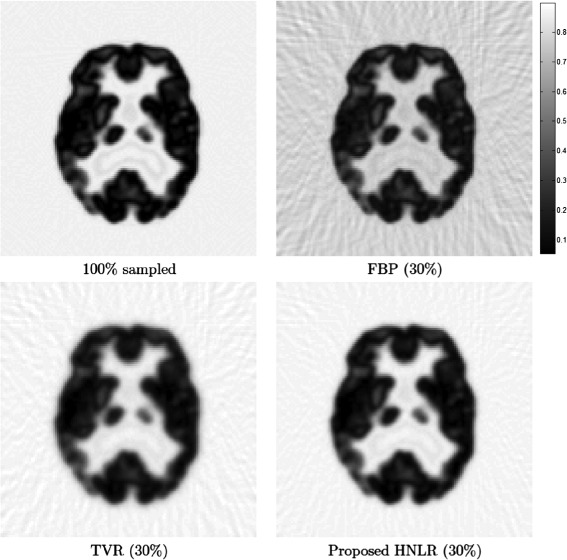
**Reconstructed tomograms from the PET3 data set using three different reconstruction methods based on 30% sampling.** The PET3 data set was acquired from a 70 year-old man with mild Alzheimer’s disease about 9 months prior to imaging. As with the reconstructed tomograms from the PET1 and PET2 data sets, it can be observed that the FBP method results in the most significant artifacts in both the background and imaged brain areas when compared to TVR and HNLR. The TVR method results in reduced artifacts but noticeable loss in functional variation differences in the imaged brain area, and the HNLR method results in the least amount of artifacts and better preservation of functional variation differences in the imaged brain area.

Figures 3, 4 and 5 shows PET tomograms from the PET data sets reconstructed at 30% sampling using the three reconstruction methods. We can see very clearly that the FBP method leads to considerable artifacts, particularly streaking artifacts, in both the background and imaged brain areas, which can affect the visibility of certain functional details in the reconstructed data. TVR provides improved reconstruction performance than FBP, in terms of reduced artifacts, but still has noticeable artifacts in the background area and detail loss in the imaged brain area, which can obscure important functional characteristics in the tomograms. Finally, the reconstructed tomogram produced using the proposed HNLR approach exhibits significantly fewer artifacts, particularly streaking artifacts, in the background area compared to FBP and TVR, while providing better functional detail preservation in the imaged brain areas in the reconstructed tomograms than TVR. The artifact reduction by both the TVR and HNLR methods within the brain region is particularly noticeable in Figure 4, where it is very clear that streaking artifacts (caused by sampling at a sub-Nyquist sampling fraction) is prominently overlaid on the entire brain region in the reconstruction produced using the FBP method (see red arrows), while they are significantly reduced in the reconstructions produced by TVR and HNLR as well as an improvement in contrast. It is also important to highlight that, within the same brain region in the HNLR reconstruction, there is an overall improvement in sharpness when compared to the TVR reconstruction (see green arrows). Figure 4 also shows two line profiles from the PET data sets reconstructed using the methods, and further illustrates the reduction in artifacts and improved contrast achieved by TVR and HNLR when compared to FBP.

**Figure 6:**
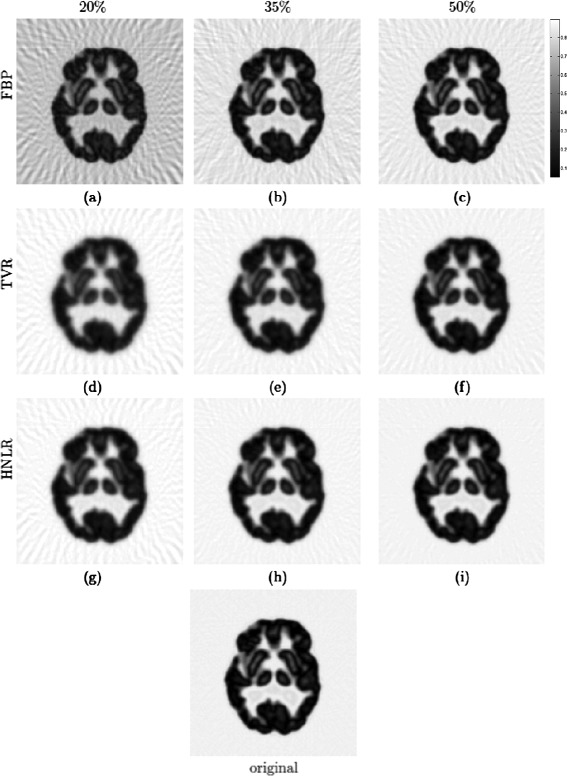
**Another set of reconstructed tomograms from the PET3 data set using three different reconstruction methods (FBP: a-c, TVR: d-f, HNLR: g-i) at 20%, 35% and 50% sampling fractions.** It can be observed that the proposed HNLR method can achieve strong reconstruction results even at relatively low 20% sparse sampling fraction, while the reconstruction results of FBP has very strong artifacts and the reconstruction results of TVR show noticeable loss in detail.

Figure 6 shows the reconstruction results of all three tested methods at different sampling fractions (from 20% to 50%). Each row shows the reconstruction results of same method, while each column shows the reconstruction results acquired at the same sampling fraction, but with different methods. It can be observed that FBP produces results that contain a large amount of artifacts when sampled at low sampling fractions. The amount of artifacts decrease dramatically when using TVR compared to FBP. However, it can be seen that the reconstruction results using TVR have noticeable loss in detail. The third row of Figure 6 shows reconstruction results of the proposed method at three different sampling fractions, which has noticeably fewer artifact residuals compared to FBP and TVR even at a 20% sampling fraction. It is important to note that while the proposed HNLR reconstruction method can help reduce artifacts caused by sampling at sub-Nyquist sampling fractions, it is limited in its performance when dealing with very low sampling fractions, as evident in Figure 6(g) where noticeable artifacts still persists.

## Conclusion

The potential use of homotopic non-local regularization (HNLR) reconstruction framework to maintaining high reconstruction quality while reducing sampling fraction in sparse PET reconstruction was studied. The proposed reconstruction framework is designed to address issues associated with artifacts and detail loss with sparse PET reconstruction. A comparative analysis using real PET data sets was performed to compare traditional FBP as well as TVR reconstruction with the proposed HNLR reconstruction method. Based on experimental results, we illustrate that promising reconstruction performance can be achieved using HNLR even at low sampling fractions. Future work includes a more comprehensive evaluative study using larger PET data sets to further validate the performance of the proposed HNLR framework, the investigation of different sparsifying transforms to study their potential for improving reconstruction quality, and the investigation of incorporating alternative representations such as multiscale texture representations and local phase [32,33] for enforcing regularization. Furthermore, we wish to investigate more advanced data terms such as the Kullback-Leibler divergence [28,29], which takes better advantage of the Poisson noise statistics of PET, as well as investigate the application of the HNLR framework for other medical imaging modalities such as diffusion weighted magnetic resonance imaging [34,35] and correlated diffusion imaging [36]. We also aim to investigate methods for automatically optimizing the parameters of the HNLR framework to obtain optimal reconstruction accuracy. Finally, we wish to investigate the effects of employing the HNLR framework for sparse reconstruction on medical image analysis tasks such as medical image annotation and contouring [37], as well as medical image registration [32,33,38-41].
